# Potential Applications of Carbohydrases Immobilization in the Food Industry

**DOI:** 10.3390/ijms14011335

**Published:** 2013-01-11

**Authors:** Fabiano Jares Contesini, Joelise de Alencar Figueira, Haroldo Yukio Kawaguti, Pedro Carlos de Barros Fernandes, Patrícia de Oliveira Carvalho, Maria da Graça Nascimento, Hélia Harumi Sato

**Affiliations:** 1 Laboratory of Food Biochemistry, Department of Food Science, College of Food Engineering, State University of Campinas (UNICAMP), Monteiro Lobato Street, 80, 13083-862, P.O. Box 6121, Campinas, SP, Brazil; E-Mails: joangelotti@gmail.com (J.A.F.); hkawaguti@gmail.com (H.Y.K.); heliah@fea.unicamp.br (H.H.S.); 2 Department of Bioengineering, Higher Technical Institute (IST), Avenida Rovisco Pais, 1049-001 Lisboa, Portugal; E-Mail: pedro.fernandes@ist.utl.pt; 3 Laboratory of Multidisciplinary Research, University São Francisco, São Francisco de Assis Av, 218, 12916-900, Bragança Paulista, SP, Brazil; E-Mail: patricia.carvalho@usf.edu.br; 4 Chemistry Department, Federal University of Santa Catarina, Florianópolis, Santa Catarina, 88040-900, Brazil; E-Mail: maria.nascimento@ufsc.br

**Keywords:** carbohydrases immobilization, amylases, invertases, inulinases, galactosidases, glucosidases, fructosyltransferases, pectinases, glucosyltransferases

## Abstract

Carbohydrases find a wide application in industrial processes and products, mainly in the food industry. With these enzymes, it is possible to obtain different types of sugar syrups (*viz.* glucose, fructose and inverted sugar syrups), prebiotics (*viz.* galactooligossacharides and fructooligossacharides) and isomaltulose, which is an interesting sweetener substitute for sucrose to improve the sensory properties of juices and wines and to reduce lactose in milk. The most important carbohydrases to accomplish these goals are of microbial origin and include amylases (α-amylases and glucoamylases), invertases, inulinases, galactosidases, glucosidases, fructosyltransferases, pectinases and glucosyltransferases. Yet, for all these processes to be cost-effective for industrial application, a very efficient, simple and cheap immobilization technique is required. Immobilization techniques can involve adsorption, entrapment or covalent bonding of the enzyme into an insoluble support, or carrier-free methods, usually based on the formation of cross-linked enzyme aggregates (CLEAs). They include a broad variety of supports, such as magnetic materials, gums, gels, synthetic polymers and ionic resins. All these techniques present advantages and disadvantages and several parameters must be considered. In this work, the most recent and important studies on the immobilization of carbohydrases with potential application in the food industry are reviewed.

## 1. Introduction

The food industry is a sector where biotechnology finds a wide application. Within this context, different groups of enzymes have potential applications in many industrial steps, including food processing. Carbohydrases represent a group of enzymes, which is among the most frequently used in the food industry. In the broadest sense, the term carbohydrase is considered to encompass diverse enzymes involved in hydrolysis and synthesis of carbohydrates, such as: amylases and invertases used in the production of syrups form starch and sucrose; β-glucosidase in the processing of fruit juices and wine, namely for flavor enhancement, and for oligosaccharide production; and glucosyltransferases for the synthesis of isomaltulose, fructosyltransferases and β-galactosidase for the production of prebiotics (Table 1) [1].

Most of the carbohydrases applied in the food industry are obtained from microbial sources, such as bacteria, yeasts and fungi, since this approach allows for maximum consistency. Microorganisms grow faster and easier than mammalian and plant cells, and the enzyme production is neither influenced by climatic conditions or seasonal changes, nor by regulatory or ethical issues related to animal slaughter or tree or plant felling. Extracellular enzyme-producing microorganisms are preferred, since this simplifies downstream processing, hence further lowering costs [1,9].

A key issue when the industrial application of an enzyme is envisaged is cost-effectiveness. This takes into consideration both the production costs and the operational lifetime of the biocatalyst. The production of industrial enzymes by genetically modified microorganisms using recombinant DNA, which has been widely implemented, has made a significant contribution to cut down production costs. This approach allows for high levels of expression and purity to be achieved, even if industrial enzymes are made available as partially purified or bulk enzymes, unlike the highly purified enzymes required for analytical or diagnostic applications. Moreover, the use of recombinant DNA technology allows for the expression of enzymes that are naturally produced by pathogenic organisms and/or organisms with low expression levels and, together with directed evolution and bioinformatic tools, paves the way for protein engineering, so that the properties of the enzyme can be optimized for specific uses [9–11]. The quest for cheaper fermentative production media, such as agro-industrial, is another strategy for lowering the costs of enzyme production [12]. Despite all this, should the enzyme be discarded after its use, the cost-effectiveness of many enzyme-based processes could be severely jeopardized. Enzyme immobilization and use of the resulting immobilized biocatalyst may provide a sound approach to resolve this [9,13,14]. As can be seen in Table 2, several carbohydrases have been immobilized using different supports (Table 2).

Using immobilized enzymes enables the reuse of the biocatalysts, since they can be easily recovered, and it allows for continuous operation, which is clearly favored in an industrial environment. Moreover, immobilization may stabilize enzymes (although this does not always occur, namely if immobilization is random, rather than properly designed [24]), make enzymes less sensitive to environmental conditions; improve the control of the reaction (*viz.* altered selectivity or specificity), avoid product contamination with the enzyme and enhance enzyme activity (although, this latter feature is less common [13,14,24–27]). A review on whether improvement in activity, specificity and selectivity as a result of immobilization anchor on realistic issues or are only artifacts has been published recently, where these matters are critically addressed [26]. The stabilization of enzymes due to immobilization can be due to several factors. Immobilization inside a porous material prevents both interfacial interaction, resulting in the presence of air bubbles, shear stress due to stirring or organic-aqueous systems and interaction with macromolecules of the enzyme extract, that otherwise may lead to aggregation, autolysis or proteolysis [28–32]. When non-porous supports are used, and enzymes are thus immobilized on the external surface of the support particles, this protective effect was mimicked through the coating of the enzyme molecules with aldehyde dextran [33]. Rigidification of enzyme structure has also been shown to result from immobilization. For that purpose, both activated synthetic carriers are especially useful. These supports thus provide a high density of attachment sites to covalently immobilize the enzyme molecule through multi-point covalent attachment to several residues placed on the enzyme surface. As a result, conformational changes promoted by agents that are involved in enzyme inactivation are minimized [24,27,34] Stabilization of multimeric enzymes (d-amino acid oxidase, β-galactosidase, alcohol dehydrogenase) may also be achieved through immobilization, by preventing the dissociation of the enzyme in its subunits. Hence, binding of the different subunits is aimed at in [24,27,35,36]. This strategy has been shown to be particularly effective fro dimeric and trimeric enzymes when anchored on densely activated supports, again, typically epoxy- and glyoxyl-based supports, which prevent dissociation and enhance the overall rigidity of the enzyme [24,35,37]. For more complex structures, *viz.* tetrameric enzymes (ampicillin acylase or catalases) or multiprotein complexes, the combination of multipoint attachment in densely activated supports and additional cross-linking was shown to allow the binding of immobilized and non-immobilized subunits, hence stabilizing such structures [24,35,37]. Penalties commonly associated with immobilization are mass transfer limitations, costs associated with immobilization and eventual loss of intrinsicality [13,14,24–26]. Several parameters are important when industrial applications of the enzymes are considered. Particular relevance is given to mechanical strength, chemical and physical stability, hydrophobic/hydrophilic character, enzyme loading capacity and cost of the support and cost of the immobilization procedure [13,14,25,38].

The present work provides an overview of recent developments on the use of immobilized carbohydrases for application in the food industry.

## 2. Enzyme Immobilization Techniques

Enzyme immobilization techniques may be performed by: (a) binding of the enzyme to a prefabricated carrier; (b) entrapping or encapsulating the enzyme in a three-dimensional polymeric network formed in the presence of the enzyme; and (c) carrier-free methodologies that rely in the cross-linking of enzyme crystals (CLECs) or enzyme aggregates (CLEAs) with bifunctional reagents to produce macroparticles [13,14,25]. Furthermore, carrier-free methodology significantly reduces the amount of non-catalytic material that is used in enzyme formulations. Eventually, a mixture of two different techniques can be used, namely, combining: (a) CLEAs with entrapment [39]; (b) cross-linking with binding to a previously formed support [40]; (c) cross-linking and entrapment [41]; and (d) adsorption and covalent bonding [42,43].

Although the immobilization of enzymes has had by far an empiric nature, a more rational approach towards this approach has been gaining relevance. This anchors in having detailed insight of the structure of the enzyme molecule (*viz.* distribution of residues available for immobilization, distribution of hydrophilic/hydrophobic zones of the enzyme, electrostatic potential of the molecule, locus of the active site), but also of the morphological and chemical characteristics of the carrier. Dedicated software has also been developed that enables the generation of predictive models of immobilization efficiency [14,44]. Enzyme binding to a carrier may be carried out by physical adsorption or by covalent attachment. In the former case, hydrogen bonds, van der Waals forces, ionic bonding or hydrophobic interactions may be involved. Adsorption is easy and cheap to perform, and negligible enzyme deactivation is typically observed as an outcome of immobilization procedures. However, changes in substrate and salt concentration, enzyme load, flow rate, temperature or pH fluctuations easily lead to desorption and, consequently, enzyme leakage. Hence, constant operational conditions are required. On the other hand, desorption of enzymes at the end of the active life of the biocatalysts enables the regeneration and re-use of the carrier [14,41,45,46].

Adsorption can be of particular interest in non-aqueous media, considering the intrinsic insolubility of the enzyme in organic solvents [13,47]. The fusion of the enzyme with a cationic, arginine-rich variant of a protein domain has been suggested in order to enhance the driving force for non-covalent binding to an anionic carrier. Furthermore, the methodology implemented also increased the selectivity of binding, since, at neutral pH, most of the proteins used displayed a negative charge. Moreover, the charge asymmetry resulting from the introduction of the residue led to a preferred orientation of binding, so that unwanted interactions between the carrier surface and active site of the modified enzyme are not favorable [45]. Bolivar and Nidetzky [45] have also shown that under selected conditions, elution of these modified enzymes could be eluted from silica materials, which are widely used for enzyme immobilization, hence allowing carrier reuse.

Enzyme immobilization by covalent binding to an insoluble support is particularly advisable when the absence of enzyme in the product stream is a strict requirement, given the stable nature of the bonds established between active amino acid residues located on the surface of the enzyme and active functional groups present at the surface of the carrier [48]. A drawback to this approach is that it often involves the use of chemicals and/or operational conditions that are hazardous for enzyme activity, features that were particularly noticeable in the early developed carriers and methods [49]. This has been overcome through the use of less aggressive techniques and supports, namely with the introduction of pre-activated supports [13,43,50]. Commercially available epoxy activated supports (*viz.* Eupergit^®^, Sepabeads^®^) were shown to provide adequate matrices for enzyme immobilization through a two step process. This involved a rapid and mild physical adsorption of the enzyme to the support, followed by a covalent binding between the adsorbed enzyme molecules and neighboring epoxide groups [14,51–53]. This first generation of epoxy supports displayed only moderate enzyme immobilization rates. Such behavior was noticed since only a small fraction of the epoxy groups on the support surface could be modified with amino groups, as a compromise between the rates of both enzyme adsorption and covalent immobilization [53]. Moreover, a high ionic strength environment was required for immobilization, often with a negative impact on enzyme stability. Again, since immobilization involved hydrophobic areas of the enzyme and hydrophobic supports, enzyme stability could be occasionally compromised [43,54]. Bearing in mind the two-step mechanism for the covalent immobilization of proteins on epoxy supports, a second generation of hetero-functional supports was thus developed. These combined two types of functional groups: one group enabling the physical adsorption of the enzymes (*viz.* by ion exchange or adsorption on immobilized metal-chelates), obtained by the modification of epoxy groups; and another group enabling the covalent immobilization of the enzymes (*viz.* epoxy groups). Enzymes can thus be adsorbed to the support through different phenomena and then covalently bound through linkage between the epoxy group and nucleophilic groups of the enzyme [42]. This broadened the range both of enzymes that could be immobilized from protein extracts and of operational conditions and avoided the need of hydrophobic supports, so that hetero-functional agarose, for instance, could be used. However, in the design of these supports, the extent of epoxy modification required a compromise for achieving high rates of both adsorption and covalent binding. Thus, the fraction of modified surface epoxy groups was relatively low, concomitantly leading to moderate overall enzyme immobilization rates. This was overcome by the use of supports, where the epoxy groups were deposited on a layer of ethylenediamine, which is covalently bound to the support (*viz.* Sepabeads EC) [50,53]. Such supports display both high fractions of amino groups and a high density of epoxy groups. The advantage of these supports over conventional and second-generation supports were highlighted while testing several enzymes (*viz.* β-galactosidase, glutaryl acylase) and assessing parameters, such as activity recovery and thermal stability [53]. A fourth generation of epoxy-based supports was developed, aiming for further control of immobilization. These supports contained a small fraction of thiol reactive groups, thiol being the most reactive nucleophiles in proteins, and a large density of epoxy groups [53]. Binding of either chemically or genetically modified enzymes can lead to a very stable biocatalyst, due to multi-point or site-directed rigidification [55]. This strategy, involving the manipulation of the enzyme surface to promote a more efficient multi-point attachment to the support through the two step process, has been implemented for other supports, such as glyoxyl-activated supports. These supports have been shown to promote stronger multi-point covalent attachment than epoxy-based supports, thus enhancing protein stabilizations [56–58]. Further developments on the design of supports allowing for multi-point covalent attachments and their combined use with site-directed mutagenesis of enzymes to further improve immobilization have been reviewed recently [59].

Entrapment/encapsulation of enzymes entails the occlusion of enzyme molecules within a polymeric network, while allowing for the flow of substrate(s) and product(s). This methodology mostly involves biocompatible materials and mild conditions, but the resulting carriers may be prone to enzyme leakage, hence pre- or post-treatment may be required. The polymeric networks can be of a dense (entrapment) or hollow (encapsulation) nature, and be made of polymers, *viz*. natural gelling polysaccharides, such as alginate, carrageenan, chitosan or pectin, proteins, such as gelatin, and synthetic polymers, such as polyvinyl alcohol, or sol-gel composites [29,34]. The latter are chemically inert silica-based materials, made out of tetraalkoxysilanes, which can be shaped on demand and display high thermal and mechanical stability. Care must be given in the methodology for producing sol-gel carriers, so as to avoid restricted diffusion of substrate to the enzyme, as a result of the low porosity of the support [13,60,61].

Cross-linking of enzymes for application in bioconversion processes relies on the formation of enzyme aggregates. CLEAs preparation usually starts with the generation of enzyme aggregates. To achieve this, a precipitant, *viz*. acetone, ammonium sulfate, ethanol or 1,2-dimethoxyethane, is added to the enzyme solution, after which the cross-linking agent is added. Usually, glutaraldehyde is used, since it is cheap and widely available. Although the chemistry of the process is not fully understood, it is established that the free amino groups of lysine residues on the surface of neighboring enzyme molecules react with oligomers or polymers of glutaraldehyde that result from inter- and intra-molecular aldol condensations. The cross-linking process is pH-dependent. CLEAs formation may also take place in the presence of additives (*viz*. magnetic nanoparticles, given polymers, bovine serum albumin) to convey smart biocatalysts, with tunable physical properties or as protective agents. Co-aggregation of bovine serum albumin (BSA) with lipase was shown to increase enzyme activity and recovery [62]. These authors identified a range of BSA concentration that allowed for an optimum level of cross-linking with glutaraldehyde increase in transesterification activity. Beyond such range of BSA concentration, there was either excessive cross-linking, due to lack of free amino groups, or an excess of amino groups available, ultimately preventing the required cross-linking of lipase molecules. The authors used the same approach for the preparation of co-aggregates of penicillin acylase and BSA, leading to a CLEA with twice the catalytic efficiency and improved thermal stability, when compared with CLEA prepared without BSA [62]. Dong and co-workers also used BSA to increase the content of lysine residues and, concomitantly, optimize the cross-linking efficiency, for the production of CLEAs of aminoacylase [63]. These authors observed an increase in activity recovery as a result of using BSA as additive in the preparation of CLEAs, since it allowed for more effective cross-linking. Still, care had to be taken on the mass ratio of gluataradehyde:enzyme, since when it increased from 0.75 to 1, a decrease in the specific activity was also observed. This behavior could be due to an increased fraction of enzyme inactivated or to tightening of CLEAs, hence enhanced diffusion resistances. Dong *et al.* also reported an increase in the thermal stability of CLEAs produced using BSA as additive when compared to CLEAs prepared without this proteic feeder. This behavior was tentatively ascribed to deficient cross-linking in the later case, resulting in release of aminoacylase from the aggregate when exposed to high temperature. On the other hand, co-aggegates of laccase with BSA displayed lower activity, but higher stability than CLEAs without BSA. The lower activity of the co-aggregates was ascribed to shielding of the active site, as well as to mass transfer limitations. The higher stability of the co-aggregates was associated to the microenvironment created by significant amounts of BSA surrounding laccase. This favored cross-linking and decreased the entropy of the enzyme denatured state [64]. A different approach to overcome a deficient cross-linking, due to a low number of reactive groups on the surface of the enzyme, was suggested by López-Gallego and co-workers [65]. These authors carried out the aggregation of glutaryl acylase in the presence of polyethyleneimine. In the process, the amino groups of the polymer are brought closer to the scarce primary amino groups on the enzyme surface, promoting their cross-linking. This led to CLEAs with enhanced thermal stability when compared to the free enzyme, due to the rigidification of the tertiary structure of the enzyme, resultant of the cross-linking.

In order to prevent CLEAs deactivation in the presence of organic solvents, Wilson and co-workers produced co-aggregates of penicillin G acylase and polyethyleneimine and dextran sulfate [66]. These polymers created an extremely hydrophilic coating of the CLEAs. The authors were able to establish that the hydrolytic activity of the polymer coated CLEAs decreased with an increase in the protein:polymer mass ratio and with the increase in the chain-length of the polymers, which they related to diffusion limitations. Polymer coated CLEAs displayed higher operational stability in the presence of organic media than simple CLEAs. Such increase, based in the half-life, could be of 16- to 26-fold, whether polymers with molecular weights either within 5000–25,000 Da or with 1,000,000 Da were used, respectively. This behavior was related to the larger polymers covering the enzyme in a more efficient manner. On the other hand, the thermal stability of CLEAs was not altered by polymer coating, which further suggested that increased stability of coated CLEAs in an organic environment is due to the hydrophilic microenvironment surrounding the enzyme, rather than to a more rigid conformation of the enzyme.

In order to overcome the relatively mechanical stability of CLEAs, the aggregates have been encapsulated in rigid polymeric materials, such as a polyvinyl alcohol-based hydrogel, LentiKats^®^ [67]. Although the encapsulation was shown to decrease by 40%, the activity of penicillin G acylase aggregates, due to diffusion limitations, in the encapsulation improved the thermal stability of the CLEA in the presence of organic solvents. This was due to a hydrophilic microenvironment surrounding the enzyme, created by the hydrogel [68].

Kim and co-workers developed a novel approach to produce enzymatic nanocomposites through the adsorption of either α-chymotrypsin or lipase in hierarchically ordered, mesocellular, mesoporous silica, followed by cross-linking with glutaraldehyde. The resulting magnetic CLEAs were shown to be easily recoverable from the reaction medium by the use of a magnet, retained their integrity, even under harsh shaking condition, unlike the nanocomposites without glutaraldehyde treated, and could be recycled for repeated batch runs without significant loss of catalytic activity [69].

## 3. Immobilized Enzymes in Syrup Production

### 3.1. Amylolytic Enzymes

**Figure 1:**
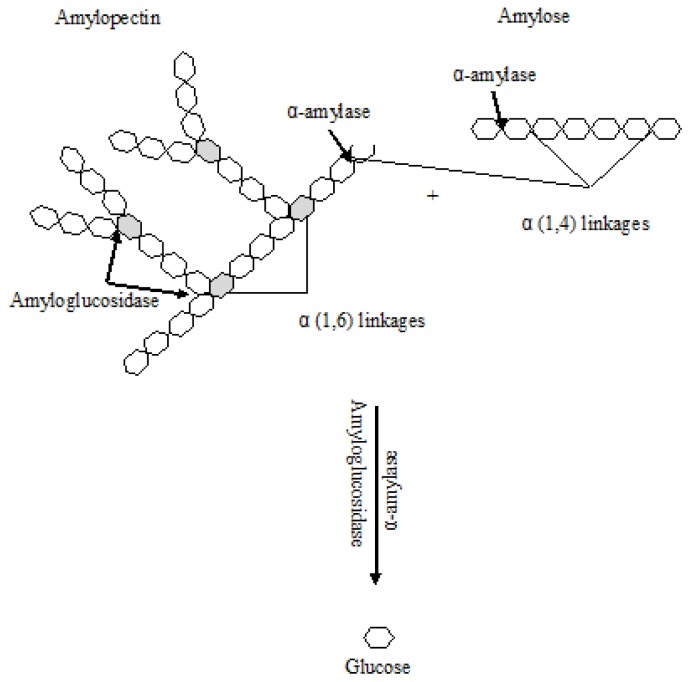
Hydrolysis of starch using different amylolytic enzymes.

Amylases compose one important group of enzymes that can be applied in the production of maltose, maltodextrin, glucose and high fructose syrups using starch as substrate (Figure 1). The production of glucose syrups involves two steps. Firstly, the starch is liquefied where the α-amylase partially hydrolyzes starch to maltodextrins and, secondly, saccharification happens where the low dextrose equivalent (D.E.) syrup is completely converted to glucose by glucoamylase [70]. Pullulanase, a starch debranching enzyme, can be also used in the later process in addition to glucoamylase [71]. Several works describe the immobilization of amylases. One particular issue that should be taken into consideration in the design of immobilized amylases is the bulky nature of the starch substrate, which may reach up to 80 MDa [72]. Hence, the small pore sizes of several commonly used supports, under 100 nm, may cause significant diffusion resistances and hinder the access of the starch molecules to the active site, hence leading to low overall reaction rates [49,72], although this is not always observed,. These can be reduced by use of matrix supports with large pore diameters [49]. This pattern was observed by Ivanova and co-workers when covalently binding amylase G from *Bacillus licheniformis* to Sperosils with pore sizes within 460 nm to 8 nm, to perform starch hydrolysis, although some deviation was observed when amylase A was immobilized [73]. Still, no mass transfer limitations were observed when the much smaller amylopectin was used as substrate. Shewale and Pandi immobilized α-amylase in a super-porous support (pore diameter about 3 μM), CELBEADS, aiming to capitalize on this matter for starch hydrolysis [74]. Still, the apparent *K*_m_ was 4.5-fold higher than that of the free enzyme, a feature the authors ascribed either to the structural changes in the enzyme occurring as a result of the covalent immobilization or still persisting diffusion limitations and steric hindrances limiting the access of the substrate to the active site. Nevertheless, immobilization enhanced thermal stability, and moreover, the immobilized enzyme retained full activity after eight batches of hydrolysis. These results also allow pointing out that modifications in the apparent affinity of the immobilized enzyme towards the substrate may not solely reflect changes in mass transfer. Also, a direct match of different works, when aiming to compare the impact of immobilization on the access to the active site of the bulky substrate, proves a bit difficult, since the actual average size of the substrate is not always referred, and this may have a clear significance.

Most of the recent studies on α-amylase immobilization use adsorption and covalent binding techniques. In the work of Singh and Kumar [75], carboxymethyl tamarind gum, a derivative of the cheap and plentiful tamarind seed polysaccharide with enhanced water solubility, initiated and catalyzed the sol–gel polymerization of tetramethoxysilane. The outcome was a monolithic silica-polysaccharide nanohybrid, upon which α-amylase was immobilized by adsorption. The resulting catalytically-active nanohybrid was thoroughly characterized through different analytical methods, *viz*. BET, FTIR, SEM, TGA and XRD, and applied to the hydrolysis of soluble potato starch to glucose syrup. The optimum pH and temperature for the hydrolysis reaction were pH 5.0 and 40 °C, respectively. The kinetic parameters, *K*_m_ (4.261 g/L) and *V*_max_ (2.55 mol mL^−1^ min^−1^), for the immobilized amylase were found favorable compared to the corresponding values obtained for the free enzyme (*K*_m_ = 6.269 mg/mL, *V*_max_ = 1.53 mol mL^−1^ min^−1^), despite the narrow size (6.1 nm) of the pores of the supports. The authors suggest that the observed changes in the kinetic parameters as a result of immobilization result from a positive distortion of the enzyme assembly. Moreover, no steric hindrances were considered to be present that restrained the access of the substrate to the active center. The enzymatic activity of the nanohybrid matrix remained stable for up to 90 days [75].

The α-amylase from *Aspergillus oryzae* was immobilized on Magnetic poly(2-hydroxyethylmethacrylate)/Cibacron blue [mPHEMA]/CB beads by adsorption. The authors observed that the adsorbed amounts of α-amylase per unit mass of magnetic beads reached a plateau value at about 1.0 mg/mL at pH 5.0. The optimum pH for catalytic activity for free and immobilized enzyme was 7.0 and 8.0, respectively, and catalytic parameters remained almost unaltered as a result of immobilization. In this case, the magnetic particles had an average pore size of 814 nm, which would contribute to reducing mass transfer limitations. The thermal stability of the immobilized enzyme was higher than the free form. After 35 days of storage at 4 °C, the free α-amylase lost all of its activity, whereas the immobilized enzyme lost approximately 27% of its activity during the same period. Moreover, enzyme adsorption–desorption cycles onto/from [mPHEMA]/CB beads were repeated up to five times. These studies showed that there was no significant reduction in the adsorption capacity of the beads, which could thus be repeatedly used in enzyme immobilization [76].

However, and given the simplicity and mild conditions of hydrogel encapsulation, research is still carried out aiming to improve this technique for application to α-amylase. An example is the recent work by Talekar and Chavare, aimed at identifying the optimum conditions for producing an α-amylase biocatalyst entrapped in calcium alginate for starch hydrolysis [77]. These authors were able to establish this using 3% (*w*/*v*) alginate, 1 M calcium chloride and 120 min of curing time; an activity recovery of 90% was obtained, which may be the result of a tighter network, which minimizes enzyme leakage. The resulting biocatalyst displayed enhanced thermal stability, and the kinetic parameters did not differ significantly from the free form, suggesting negligible mass transfer resistances and no structural enzyme modifications as a result of immobilization. Reusability proved, however, a bit disappointing, since after 10 cycles, the activity decreased to about 35% of the initial value. Moreover, a reaction-diffusion mathematical model has been recently developed to simulate starch hydrolysis promoted by calcium alginate entrapped α-amylase, in a process carried out in a packed-bed reactor [72].

Carpio *et al.* [78] studied the glucoamylase immobilization and its subsequent use in cassava starch hydrolysis. The enzyme was immobilized onto chicken bone particles, and some parameters, such as pH, ionic strength, particle size and enzyme load, were evaluated. 270 units of glucoamylase were adsorbed per gram of support, and the optimal temperature and thermal stability of immobilized enzyme were only slightly different from those of the free enzyme, while the optimal pH became more acidic by about one unit. The immobilized biocatalyst was used for the production of high glucose syrup from liquefied cassava starch. The process was implemented at bench scale in a batch process using a stirred-tank reactor. Similar conversions to those achieved with soluble enzymes, and corresponding to 98 DE, were reached, until the third batch, and over 90 DE were observed until the 25th batch.

The glucoamylasefrom *Aspergillus niger* was immobilized onto functionalized magnetic SBA-15 (FeSBA-15) as a regenerated support through metal-ion affinity interactions in the work of Zhao *et al.* [79]. The authors observed that the thermal stability of immobilized glucoamylase was much better than the soluble form. This behavior was ascribed to the enhanced enzyme rigidity conveyed by the enzyme binding to the carrier, thus preventing unfolding and conformation transition of the enzyme at high temperatures.

Pullulanase from *Bacillus acidopullulyticus* was covalently immobilized onto the hydrophobic synthetic macroporous resin Duolite XAD761 through the formation of Schiff bases [80]. The authors optimized the immobilization procedures, namely the incubation time and glutaraldehyde concentration for activation of the resin and enzyme loading and coupling time. As an outcome of immobilization, the optimum pH for activity was shifted from 5.0 to 5.5, and the temperature optimum for activity increased from 50 to 60 °C, as compared to the free form. The immobilized biocatalyst also displayed enhanced thermal stability as compared to the free enzyme. The substrate specificity was not altered as a result of immobilization, but the *K*_m_ values for the substrates assayed (pullulan, soluble starch and dextran) increased. The authors ascribed this feature to steric hindrances or to conformational changes of the enzyme molecules as an outcome of immobilization. The immobilized catalyst was reused throughout 35 consecutive cycles for pullulan (0.44% *w*/*v* solution) hydrolysis. The percent hydrolysed was reduced to about 80% after 12 cycles, and after 35 cycles, it was reduced to a little under 30% [80]. The same immobilized biocatalyst was also used for the development of a continuous flow reactor, again using pullulan hydrolysis as a model system. Continuous operation was carried out throughout 32 days. After 15 days of operation, 80% hydrolysis was observed, but this gradually decreased to about 50% after 31 days of operation. At this point, the activity of the immobilized biocatalyst was also shown to have achieved its half-life [81].

Recently, Talekar *et al.* [82] prepared spherically-shaped magnetic CLEAs of α-amylase by cross-linking enzyme aggregates with amino-functionalized magnetite nanoparticles. The inclusion of magnetic particles eased biocatalyst recovery from the reaction media. Moreover, it allowed for full recovery of activity during immobilization, whereas the use of simple CLEAs only allowed for 45% activity recovery. Both simple CLEAs and magnetic CLEAs displayed a shift in optimal pH towards more acidic values, and the optimal temperature of magnetic CLEAs was higher than that of simple CLEAs and of free enzymes. Moreover, the enzyme affinity to the substrate increased as a result of immobilization in either CLEA. Both storage and thermal stability of α-amylase were enhanced as a result of immobilization in magnetic CLEAs, which were also reused for six cycles without any decay in the initial activity.

### 3.2. Invertases

One important type of sugar syrup is the inverted sugar syrup. It is broadly used in the food industry, since it produces denser solutions compared to sucrose, showing lower crystallization and growth of microorganisms. Furthermore, when compared to sucrose solution, inverted sugar syrup is 20% sweeter and has a lower freezing point and higher affinity for water [83]. This syrup can be obtained through the hydrolysis of sucrose syrups with invertase. In the use of the enzyme, the immobilized form is preferred, since it presents several advantages, such as reusability [84]. Some recent works focus on covalent binding techniques.

In the work of Cadena *et al.* [85], polyurethane rigid adhesive foam was used to covalently immobilize invertase for application in an enzymatic bioreactor. Immobilization increased the enzyme affinity to the substrate, as suggested by the decrease in *K*_m_ from 61.2 mM to 46.5 mM. On the other hand, a 10-fold decrease in the turnover rate resulted from immobilization, possibly a consequence of diffusion restriction of the substrate to the immobilized biocatalyst. The immobilized enzyme retained 50% of the initial activity after eight months of storage at 4 °C, and no microbial contamination was observed. The best results for production of invert sugar syrup under operation of the packed bed were obtained with an up-flow rate of 0.48 L/h, leading to an average conversion of 10.6% h^−1^ at a feeding rate of 104 h^−1^.

The extracellular thermostable invertase from *Aspergillus awamori* was immobilized on acetic acid-solubilized chitosan by covalent binding using glutaraldehyde. This enzyme preparation was efficiently and continuously applied in a packed bed reactor to the production of high-fructose syrup from sucrose. At 50 °C and pH 6, an extract initially containing 139.2 g/L total sugar with 78.6 g/L sucrose at a flow rate of 17 mL/h was applied, resulting in a conversion factor of 0.95 and a fructose content in the syrup of 69 g/L [86].

Invertase was also covalently immobilized on glass–ceramic support (GCS). The immobilized biocatalyst was shown to retain 100% of the initial activity after nine reuses. The optimum pH and temperature for enzyme activity were not affected by immobilization, but the *K*_m_ increased 13.5 times, suggesting mass transfer resistances. Yet, when a packed bed reactor was operated in an alternate flow regime (alternation of down-flow and up-flow), it outperformed the up-flow and down-flow regime when the time course of sucrose hydrolysis was considered [87,88].

Yeast invertase was also covalently bound with glutaraldehyde to activated carbon previously treated with urea and dimethyl formamide. The optimum pH for activity was shifted from 4.0 to 6.0 as a result of immobilization, whereas the temperature optimum decreased from 50 °C to 30 °C. The maximum activity for both free and immobilized enzyme was observed for a sucrose concentration of 625 mM [89].

Cell wall invertase modified with glutaraldehyde was entrapped in calcium alginate beads. Immobilization was shown to have broad pH and thermal stability, as well as a broader pH optimum (4.0 to 5.5) and temperature optimum (55 to 70 °C), as compared to the free form (pH between 4.0 and 5.0 and temperature of 60 °C). Continuous operation with a packed-bed reactor allowed for 95% inversion of 60% (*w*/*w*) sucrose solution and for a productivity of 3844 kg of inverted sugar per kg of immobilized biocatalyst after one month of operation [90].

Invertase was also entrapped in polyvinyl alcohol-alginate beads treated with sodium sulfate for the hydrolysis of liquid pineapple waste. The temperature optimum for activity remained unaltered at 50 °C, but the pH optimum was shifted from 5.0 to 4.0 as an outcome of immobilization. This feature was ascribed to the higher proton concentration inside the beads as compared to the bulk solution, resulting from the dissolved boric acid present in the beads. The apparent affinity of the enzyme to the substrate increased significantly, due to favored mass transfer effects, and the maximum reaction rate increased, as a result of enhanced enzyme-support affinity. The beads were effectively used in 14 cycles for the full hydrolysis of 1% (*w*/*v*) sucrose solution and were stored at 4 °C for 60 days with no decay in activity. Moreover, the immobilized invertase successfully converted 91.4% of sucrose present in pineapple waste to glucose [91].

Porous CLEAs of invertase have recently been prepared. The process was carried out by using starch as a pore-making agent to a crude invertase solution, promoting co-precipitation with ammonium sulfate, and cross-linking the co-precipitate with glutaraldehyde. The kinetic parameters for sucrose hydrolysis remained unaltered as a result of immobilization, suggesting no mass transfer limitation nor deleterious effects on enzyme structure [92].

### 3.3. Inulinases

Inulin is a polyfructan found in many types of plants, and it is recognized as a source for the production of both ultra-high fructose syrup [10] and inulooligosaccharides [93]. This is very relevant, since fructose presents GRAS (Generally Recognized as Safe) status. Inulin is easily hydrolysed by exoinulinase (-d-fructan fructanohydrolase, E.C. 3.2.1.80) and endoinulinase (2,1-d-fructan fructanohydrolase, E.C. 3.2.1.7). Exoinulinase hydrolyzes inulin by splitting off the terminal fructosyl units by cleaving the glycosidic linkages to the polysaccharide moiety. On the other hand, exoinulinases were distinguishable from endoinulinase by their ability to hydrolyze sucrose [94]. Hence, fructanohydrolase from microbial sources plays an important role in the hydrolysis of inulin for its commercial production.

The enzymatic process is preferred over the chemical hydrolysis, due to the drawbacks, like production of unwanted by-products and color-forming compounds [95]. Santa *et al.* [96] used sol–gel immobilized inulinase for the hydrolysis of inulin to fructose. The authors obtained porous xerogel particles with dimensions in slight excess of 10 μM and an immobilization efficiency of roughly 80%. According to the estimated kinetic parameters, the immobilization in sol–gel did not alter the native enzyme conformation, yet the entrapment resulted in mass transfer limitations. Regarding the operational stability, the sol-gel immobilized biocatalyst was used in more than 20 consecutive batches of 24 h without a significant decrease in product yield. These results could be considered very interesting, regarding industrial application of enzymes, compared to free enzymes that cannot be reused.

Singh *et al.* [97] reported the immobilization of the partially purified extracellular exoinulinase from *Kluyveromyces marxianus* YS-1 on Duolite A568. The biochemical characteristics of the immobilized enzyme were slightly different from the soluble enzyme. The optimum pH for enzyme activity, 5.5, was not altered as a result of immobilization, whereas the latter led to a shift in the optimal temperature from 50 °C to 55 °C. The immobilized biocatalyst retained more than 90% of its original activity after incubation for 3 h at 60 °C. The activity of the free enzyme was reduced to 10% under the same conditions, indicating an improvement in the thermal stability of the biocatalyst after immobilization. When the immobilized biocatalyst was applied in four-hour batch runs for the preparation of high fructose syrup from raw and pure inulin, fructose yields of 39.2 and 40.2 g/L were reported. When recycling the biocatalysts, the percent of hydrolysis of a 5% (*w*/*v*) inulin solution decreased from a little under 85% to close to 60% after 10 cycles. The percent of hydrolysis decreased henceforth at a lower pace to attain about 40% after 55 cycles. The same immobilized biocatalyst was used in a continuous flow packed-bed reactor for the hydrolysis of a 5% (*w*/*v*) solution of inulin at 55 °C, with a residence time of 1.25 h. After 21 days of operation, the percent of hydrolysis and the volumetric productivity decreased from 100% and 44.5 g/L/h to 84% and 37 g/L/h, respectively. The reactor was operated for a total of 75 days, where the percent of hydrolysis was 50% and the volumetric productivity had decreased to about 20 g/L/h. The reactor was also operated for the hydrolysis of raw inulin, but it remained operational for only 11 days [98].

Basso *et al.* [44] carried out the rational immobilization of inulinase on Sepabeads EC-EP (oxirane group) and EC-HA (amino group), based on homology modeling, docking and molecular dynamics. The percent of bound protein was slightly higher in the latter (82%) than in the former (77%) and so was the activity, with 285 U/g_dry support_ for EC-HA support and 205 U/g_dry support_ for EC-EP support. Accordingly, the former displayed higher reaction rates for the hydrolysis of a 1% (*w*/*v*) inulin solution than the latter. However, oxirane-based supports allowed for higher final fructose yield (90%) than the amino-based supports. Inulinase immobilized in the oxirane-based supports was then used to compare the performance of a fluidized bed reactor, operating in a closed cycle configuration, with that of a batch reactor for the hydrolysis of a 1% (*w*/*v*) inulin solution [99]. The authors were able to establish that in a range of flow rates within 1.6 and 2.4 L/h, external mass transfer effects were not significant, reaction kinetics being, instead, the controlling step. The performance of the fluidized bed reactor was marginally lower than that of the batch reactor, since after seven hours of operation, the former produced 5.4 g/L fructose, compared to 6.4 g/L fructose for the latter.

The immobilization of inulinase of *K. marxianus* var. *bulgaricus* was performed by entrapment in gelatin followed by cross-linking with glutaraldehyde. The optimum pH for immobilized inulinase was 3.5, and the optimum temperature was 60 °C [100]. Ettalibi and Baratti [101] have immobilized commercial inulinase preparation onto glass beads of different porosities by amination of glass beads with 3-aminopropyl triethanoxysilane, activation with glutarldehyde and incubation with inulinase solution. The half-life of the immobilized enzyme was 350 days at 50 °C with 2 M sucrose solution. The immobilized cell system of *Kluyveromyces marxianus* had up to 85% residual activity and the immobilized enzyme was thermostable at 65 °C [102]. The residual activity in glutaraldehyde-treated beads after five cycles was up to 83%, which was much higher than that of untreated beads (39%). Enhanced recovery of *K. marxianus* inulinase was made using anionic resin Streamline DEAE, and a maximum adsorption of about 1428 U/mL was recorded [103].

Continuous production of oligofructose syrup from Jerusalem artichoke juice using immobilized *A. niger* endoinulinase on chitin has been reported [104]. The enzyme was covalently bound to the carrier chitosan using glutaraldehyde with an activity recovery of 66%. Immobilization leads to a shift in pH optimum from 4.5–5.0 to 5.5–6.0. Such shifts in pH optima after immobilization have been reported in other inulinases as well [100,101]. Immobilized endoinulinase also showed a higher temperature optimum of 65 °C than free enzyme (60 °C). Such increase has also been noticed in temperature optima of an immobilized preparation of endoinulinase from *Pseudomonas* 200 sp. and *A. ficuum* by 2.5 °C and 10 °C, respectively [101,105]. On the other hand, the affinity of the enzyme towards inulin was not affected by immobilization. Both free and immobilized enzyme preparations were stable for one year, when stored at 4 °C. Under continuous hydrolysis of inulin substrates, *viz*. Jerusalem artichoke juice or dahlia inulin, in a packed-bed reactor operating at 60 °C and pH 5.5, a half-life of 48 days was estimated. Substrate solutions in a range within 2.7% (*w*/*v*) to 25% (*w*/*v*) were fed to the column. FOS effluent was composed mostly of oligofructose molecules with a degree of polymerization (DP) between 3 and 7, suggesting that fructan molecules of a higher degree of polymerization (DP8 and more) were hydrolyzed. The DP was influenced both by substrate nature and concentration. Typically, an increase in the latter led to a decrease in the relative amount of products with DP3 and an increase in the relative amount of products with higher DP.

## 4. Immobilized Carbohydrases in the Beverage Industry

Many criteria should be adopted to choose supports for use in industrial processes, such as atoxicity (food grade), low cost and ready availability, ease of use, chemical inertia (under conditions of use), non-biodegradability and, lastly, the ability of the support to react either directly or via a simple and inexpensive activation method [106].

Several precursors of the aromatic components of wines, juices, musts and other alcoholic drinks are monoterpenes (geraniol, nerol, citronellol, linalool, α-terpineol, *etc.*) in di-glycosidic form. They contain β-d-glucopyranose bound directly to aglycon and/or other sugars, including α-l-rhamnopyranose and α-l-arabinofuranose [107].

These conjugate compounds are not volatile and are generally soluble in water, being perceived only by the consumer’s olfactory mucosa following removal of the carbohydrate moiety, by acid or enzymatic hydrolysis, that releases the volatile part (terpenols, sesquiterpenes, nor-isoprenoids, *etc.*) [108]. Glycosidases are used to increase the aroma of wines and other juices by sequential hydrolysis of the glycosides bound to the volatile compounds [109].

Spagna *et al.* [110] immobilized several glycosidases (β-d-glucopyranosidase, α-l-arabinofuranosidase, α-l-rhamnopyranosidase), purified from an *Aspergillus niger* enzyme preparation by entrapment in chitosan gels and subsequent cross-linking with glutaraldehyde. The authors aimed to assess the feasibility of applying the immobilized biocatalyst in the wine-making and fruit-juice processing industry. The authors tested the addition of various agents in order to improve physical and mechanical properties of the gel, decrease enzyme leakage and increase the immobilization yields and the operational stability. The best results were achieved using as additives gelatin and silica gel. The immobilized glycosidases were used to increase the aroma in a model wine solution. The authors established that both free and immobilized enzymes led to a four-fold increase in the concentration of several of the more fragrant volatile compounds found in wine, particularly in *Moscato* wine.

Su *et al.* [111] immobilized β-glucosidase on alginate by combining cross-linking with entrapment and, again, cross-linking. After optimization of the immobilization conditions, an activity recovery of β-glucosidase of 46.0% was obtained. The authors investigated the properties of the immobilized β-glucosidase. The optimum pH remained unaltered, while the optimum temperature was 45 °C, which was 10 °C lower than that of free enzyme. This particular feature can, however, be taken advantage of in tea beverage processing. Thus, the treatment with immobilized β-glucosidase can be carried out at a lower temperature than with the free enzymes, hence the high temperature, resulting in brownness of the tea infusion, can be avoided. Immobilization also resulted in an enhancement in the thermal stability. Moreover, the immobilized enzyme was more stable under extreme pH conditions. The *K*_m_ value for immobilized β-glucosidase was estimated to be 1.97 × 10^−3^ mol/L, about 5-fold lower than that of the free enzyme, which was ascribed to structural changes in the enzyme structure as an outcome of immobilization. The feasibility of using the immobilized enzyme as an aroma-enhancer of tea beverages was also assessed. The results showed that in tea beverages processed with immobilized β-glucosidase, the total amount of essential oil in green tea, oolong tea and black tea increased by 20.69%, 10.30% and 6.79%, respectively. Immobilization increased the storage stability of β-glucosidase, since 73.3% activity retention was observed after 42 days’ storage at 4 °C, whereas for the free enzyme, only 5.1% activity retention was observed after 20 days’ storage. Moreover, the immobilized enzyme displayed a residual activity of 93.6% after being repeatedly used 50 times.

Figueira *et al.* [112] screened various supports for the immobilization of a partially purified extract of β-glucosidase from *Aspergillus* sp. The immobilization in sol-gel and in Lentikats allowed the higher activity retention after immobilization and was thus further characterized. Immobilization did not alter the pH/activity profile, whereas the temperature/activity profile was improved when sol-gel support was assayed. Both thermal and pH stability were improved as a result of immobilization. An increase in the apparent *K*_m_ (Michaelis constant) was observed following immobilization, suggesting diffusion limitations.

Pectic enzymes have long been used in the beverage industry to increase juice yield and to clarify juices [113]. An example is pectinlyase (PL, EC 4.2.2.10), which has received growing attention for its potential application in the food processing industry, due to its ability to depolymerize pectins in a single enzyme process, doing away with the need to use pectinesterase (PE, EC 3.1.1.11), and polygalacturonase (PG, EC 3.2.1.15). The latter process releases methanol and leads to the formation of colloidal precipitates in the system, between the de-esterified pectin and the endogenous calcium ion [114,115].

Spagna *et al.* [106] used immobilized pectinlyase (PL, EC 4.2.2.10) for the depectinization of fruit juice. The authors performed the immobilization on three synthetic polymers: Eupergit C, Nylon 6 activated with glutaraldehyde and XAD7 activated with trichlorotriazine. Suitable activity results were obtained only with activated Nylon 6 and XAD7 (110 and 335 U/g, respectively). An increase in stabilization was obtained by the rigidification of the enzyme structure through cross-linking with glutaraldehyde. The pH profile remains unaltered, however, the optimum temperature of the immobilized enzyme was higher (8–10 °C) than that of the free enzyme.

Alkorta *et al.* [116] immobilized the enzyme pectin lyase [PNL, poly(methoxygalacturonide) lyase; E.C. 4.2.2.10] from *Penicillium italicurn* by covalent binding to Nylon 6 in order to compare physical-chemical and kinetic properties of the free and immobilized enzymes. The authors evaluated the optimum conditions for the immobilization process, kinetic parameters and pH and temperature behavior of the enzyme. The pH activity curve of the immobilized enzyme shifted toward a low pH compared with that of the soluble one. Similarly, the immobilized PNL was more stable at lower pH values than the free enzyme. The immobilization caused a marked increase in both thermal and storage stability of the enzyme. No loss of activity was observed when the immobilized enzyme was used for 12 consecutive cycles of operation. The decrease in viscosity of pectin solutions processed with immobilized PNL was lower than that of solutions processed with free PNL. When fruit juices were used, however, the decrease in viscosity throughout processing was as marked as that observed when the free enzyme was used to clarify pectin solutions. Given the results obtained, the authors suggested that Nylon-immobilized PNL provided a promising tool for the clarification of fruit juices at 40 °C and an approximate pH of 3.0.

## 5. Immobilized Carbohydrases in the Production of Prebiotics

### 5.1. Galactooligosaccharides

Galactooligosaccharides (GOS) are an example of functional foods. GOS are prebiotic, because they are not digested by humans or other animals, and selectively increase the beneficial microflora of the intestine, leading to health benefits. GOS can be synthesized by highly specific glycosyltransferases, which use sugar donors containing a nucleoside phosphate or a lipid phosphate remaining group. However, these enzymes are barely available, are extremely expensive and require specific sugar nucleotides as substrates. Therefore, they are not used in realistic, cost-effective processes for GOS production [117]. Thus, GOS molecules are typically synthesized by the enzymatic activity of β-galactosidase on lactose in a reaction known as transgalactosylation. The composition of GOS produced from lactose by β-galactosidase usually has the structure Gal_n_–Glc, where *n* indicates the degree of polymerization (DP) and is typically within 1–5. The process is kinetically controlled and involves competition between hydrolysis and transgalactosylation. The former, which is thermodynamically favored and leads to the production of d-galactose and d-glucose, competes with the transferase activity, which leads to the production of the complex mixture of Gal_n_–Glc saccharides. Knowledge of the time course of the reaction or of lactose conversion is needed to establish when the maximum yield of a given product is achieved. The process is quite complex, and the output clearly depends on the origin of the enzyme and on its properties [117,118]. Several models have been proposed to describe oligosaccharide synthesis and simultaneous lactose hydrolysis, ultimately pointing for the need of a high initial lactose concentration, *viz.* 40% *w*/*v* and above [6,119–122]. The recent findings of Vera and co-workers established that an increase in the initial lactose concentration only influences positively the maximum product yield, provided the lactose remains dissolved [6]. These authors also highlight the complex interaction between temperature and initial lactose concentration in the reaction of synthesis. Gosling and co-workers also produced insight on the phenomenon underlying the need for the highest possible lactose concentration to achieve high GOS levels [123]. Given the results obtained in their work, these authors suggested that such an outcome is due to increases in the reactions that lead to oligosaccharides instead of decreases in the competing reactions, which degrade oligosaccharides.

Gaur *et al.* [124] immobilized *Aspergillus oryzae* β-galactosidase by three different techniques: adsorption on Celite, covalent coupling to chitosan and aggregation by cross-linking (CLEAs). No significant changes in temperature and pH optima resulted from immobilization, however, an increase in *K*_m_ was observed, as compared to the free form. Immobilization on chitosan gave the maximum enzyme activity yield and oligosaccharide synthesis. Immobilization enhanced the thermal stability of both chitosan immobilized enzyme and CLEAs. When a 20% (*w*/*v*) lactose solution was used as substrate, the chitosan-immobilized enzyme led to a maximum oligosaccharide yield, corresponding to 17.3% of the total sugar, whereas the use of the free enzyme only allowed an oligosaccharide yield corresponding to 10.0% of the total sugars, after 2 h of operation at 40 °C. On the other hand, CLEAs proved instead effective in lactose hydrolysis, yielding 78% monosaccharide after 12 h of operation. Galactooligosaccharides were continuously produced using lactose and β-galactosidase from *Bullera singularis* ATCC 24193 immobilized in Chitopearl BCW 3510 beads [125]. No activation or cross-linking agents were used before or after immobilization. The preparation was used directly for continuous reaction in a packed bed reactor, yielding 55% (*w*/*w*) oligosaccharides and a productivity of 4.4 g/L h, from a 10% (*w*/*v*) lactose solution during a 15-day operation. Batch productivity was 6.5 g galactooligosaccharides /L h from a 0.3% (*w*/*v*) lactose solution.

Magnetic polysiloxane-polyvinyl alcohol proved to be an adequate support for the immobilization of *Aspergillus oryzae* β-galactosidase and its concomitant application on galactooligosaccharide production using lactose as substrate [121]. The support was easily recovered by applying a magnetic field, which contributed to achieving the retention of 84% of the initial enzyme activity after 10 reaction cycles. The galactooligosaccharides production was performed at temperatures varying from 30 °C to 60 °C and pH from 3.5 to 5.5, with either free or immobilized enzyme preparations, yet GOS production remained relatively unaltered despite such variations of operational condition. Magnetite particles obtained by coprecipitation of Fe^2+^ and Fe^3+^ and coated with polyaniline were activated with glutaraldehyde in order to covalently immobilize *Aspergillus oryzae*β-galactosidase [126]. The methodology allowed for the immobilization of 2.04 mg of enzyme per g of support, which the authors claimed to be the best of the values reported in the literature. This magnetic enzymatic derivative was able both to hydrolyze lactose into glucose and galactose and to produce tri- and tetra-galactosides from lactose by transgalactosylation, and, overall, its performance was akin to that of the free enzyme. Thus, for lactose concentrations up to 100 g/L, the specific activities of both forms were similar, but for higher lactose concentrations, the initial specific reaction rate of the immobilized enzyme was affected as lactose concentrations increased. GOS production by both forms of the enzyme was unaffected within 30 to 60 °C. The immobilized biocatalyst was recycled 10 times, at 25 °C and using a 20% (*w*/*v*) lactose solution in each cycle. At the end of the final cycle, the activity of the immobilized biocatalyst was 85% of the initial value. Another magnetic type of support was used for the covalent immobilization of *Kluyveromyces fragilis* β-galactosidase [127]. The magnetic nanobeads were prepared from glycidyl methacrylate, ethylene glycol dimethacrylate and hydroxyethyl methacrylate, via emulsifier-free emulsion polymerization. Binding to the enzyme molecules was formed through the epoxy groups on the surface of the beads. The maximum amount of enzyme attached to the support was of 145.6 mg/g, corresponding to an activity recovery of 72.6%. The immobilized enzyme displayed high catalytic activity for GOS synthesis and produced a total of 2.240 g GOS per gram of immobilized enzyme during 10 consecutive batch reactions. After these cycles, the immobilized biocatalyst retained 81.5% of its original activity.

A novel method of enzyme immobilization using *Aspergillus oryzae* β-galactosidase involving polyethyleneimine-enzyme aggregate formation and growth of aggregates on individual fibrils of cotton cloth leading to multilayer immobilization of the enzyme was developed by Albayrak and Yang [128]. A large amount of enzyme was immobilized, 250 mg/g support, with about 90%–95% efficiency. A maximum galactooligosaccharides production of 25%–26% (*w*/*w*) was achieved at near 50% lactose conversion from 400 g/L of lactose at pH 4.5 and 40 °C. Tri- and tetra-saccharides were the major types of galactooligosaccharides formed, accounting for about 70% and 25% of the total galactooligosaccharides produced in the reactions, respectively. A marked increase in the thermal stability of the immobilized enzyme was also observed, as compared to the free form. The half-life for the immobilized enzyme on cotton cloth was close to one year at 40 °C, but only 21 days at 50 °C. The immobilized biocatalyst enabled the GOS production of 26% (*w*/*w*) of total sugars, from a feed lactose solution of 40% (*w*/*v*) lactose at pH 4.5 and 40 °C, at 50% lactose conversion. The production of GOS from lactose was also carried out using β-galactosidase from *Aspergillus oryzae,* immobilized on a low-pressure plasma-modified cellulose acetate [129]. The novel method developed for multilayer enzyme immobilization involved the formation of a polyethyleneimine (PEI)-enzyme aggregate and concomitant growth on a cellulose acetate membrane. An enzyme load of 9.97 g/m^2^ membrane was immobilized with 66% efficiency. The half-life of the membrane immobilized enzyme was roughly one month at 30 °C, although this decreased to 60 hours at 60 °C. The maximum GOS production of 27% (*w*/*w*) was obtained at 70% lactose conversion from a 32% (*w*/*v*) lactose at pH 4.5 and 60 °C.

GOS were mostly composed by trisaccharides, as these accounted for roughly 75% of the total GOS. GOS production was also achieved using β-galactosidase from *Talaromyces thermophilus* CBS 236.58 immobilized onto Eupergit C, both under batchwise and a continuous mode of operation, the latter in a packed-bed reactor [130]. Maximum yields of GOS of 12, 39 and 80 g/L was obtained for initial lactose concentrations of 5%, 10% and 20% (*w*/*v*), respectively, for batch conversion experiments. Under continuous operation, a maximum GOS concentration of about 50 g/L was obtained with a dilution rate of 0.375 h^−1^ in a packed-bed reactor, for an initial lactose concentration of 20% (*w*/*v*). The composition of the GOS mixture was also shown to be influenced by the mode of operation. Thus, when the continuous packed-bed was used, more trisaccharides and less disaccharides were formed, as compared to batch operation. Several other papers have been published, focused on the use of different reactor configurations for GOS production, using immobilized β-galactosidase. Thus, packed-bed reactors containing immobilized β-galactosidase from *Aspergillus candidus* adsorbed onto D113 resin and entrapped in calcium alginate [131], and *Aspergillus oryzea* immobilized on cotton cloth [132] displayed GOS productivities of 87 and 106 g/L h, respectively. It has also been reported that an improved method developed by the latter authors for β-galactosidase immobilization in cotton cloth led to a productivity of 6000 g/L h [128]; GOS production in a continuous stirred tank reactor using *B. circulans* β-galactosidase immobilized in controlled pore silica gel. The yield and the productivity in GOS were 1.7- and 1.9-fold higher, respectively, when compared to those using free enzymes [133]. Nakkharat *et al.* [134] developed an ultrafiltration membrane reactor containing *Talaromyces thermophilus* β-galactosidase, which allowed for a 5.6-fold increase in GOS productivity, when compared with the free enzyme,

### 5.2. Fructooligosaccharides

Fructooligosaccharides (FOS) are also prebiotic substances, calorie-free and noncariogenic sweeteners. FOS stimulate the growth of bifidobacteria and have been claimed to contribute towards the prevention of colon cancer and to reduce cholesterol, phospholipid and triglyceride levels in serum. FOS consist of a mixture of fructose oligomers, typically with two or three fructose units bound to the β-2,1 position of sucrose, and they are mainly composed of 1-kestose (GF_2_), 1-nystose (GF_3_) and 1-fructofuranosyl-nystose (GF_4_). They are found in several vegetables or natural foods, however, the FOS are produced commercially through enzymatic synthesis from sucrose by microbial enzymes with fructosyltransferase activity [135]. The definition of transfructosylation still involves some controversy. As summarized recently, while some authors classified this reaction as that catalyzed by β-fructofuranosidase or invertase, others classified transfructosylation as a β-d-fructosyltransferase reaction [136]. When the latter is considered, fructosyltransferase activity is observed at high sucrose concentrations, whereas at low sucrose concentration hydrolytic activity is predominant. Thus, an array of disproportionate reactions leads to the formation of FOS and glucose, as a by-product from the fructosyltransferase activity, while fructose and glucose are formed as result of the hydrolytic activity [137,138]. FOS are produced according to a chain reaction, where two molecules of sucrose produce one molecule of GF2 and one molecule of glucose. Two molecules of GF_2_ react to produce one molecule of GF_3_ and one molecule of sucrose. Two molecules of GF_3_ react, leading to one molecule of GF_4_ and one molecule of GF_2_. One molecule of GF_3_ is also hydrolyzed to GF_2_ and one molecule of fructose. A predictive model, which includes substrate and glucose and fructose inhibition, was developed and provided a nice fit to the experimental data for FOS production from sucrose using a fructosyltransferase from *Rhodotorula* sp. A Michaelis–Menten behavior was observed, with substrate inhibition at high sucrose concentrations (up to 70% *w*/*v*) alongside with glucose competitive inhibition related to sucrose, GF_2_ and GF_3_ uptakes. Inhibition was also noticed at high fructose concentrations (over 50%). Hydrolyzing activity over GF_3_ was also identified [134].

There are different techniques to immobilize enzymes or whole microbial cells to produce FOS. In particular, encapsulation of the cells and enzymes is widely used. Cell entrapment in calcium alginate has been quite favored. This particular approach was implemented by Ning *et al.* [139] for the entrapment of *Xanthophyllomyces dendrorhous* cells, because of the relative ease of cell separation from the fermentation broth and the strong tolerance to high concentrations of substrates and products. The cells were mixed with 2% sodium alginate solution, and the cell-alginate mixture was extruded dropwise through a needle into a 0.2 M CaCl_2_ solution. The resulting beads were kept in CaCl_2_ solution at 4 °C for 24 h before use. The FOS procution process anchored in the use of this immobilized biocatalyst allowed for a maximum FOS yield of 236 g/L after 15 h of reaction. *Aspergillus japonicus* mycelia immobilized in calcium alginate beads were also used for the production FOS [140]. The mycelia of the strain were held in 1.5% glutaraldehyde solution at 4 °C for 24 h before entrapment with calcium alginate. FOS production was performed in a batch process in a 2-L bioreactor, and the optimum pH and temperature for synthesis were 5.0–5.6 and 55 °C, respectively. Maximum production was achieved using 5.75% (cellular weight/volume) of mycelia and 65% sucrose solution (*w:v*) for four hours of reaction, at which point 61.28% of the total FOS contained 30.56%, 26.45% and 4,27% of GF_2_, GF_3_, and GF_4_ respectively. The immobilized biocatalyst was used in 23 consecutive batches with no significant loss of activity, suggesting the potential for large scale applications. *Aureobasidium pullulans* was immobilized in calcium alginate beads and used for continuous production of fructooligosaccharides using a packed-bed reactor [141]. For the immobilization procedure, a cell suspension, containing 4% (*v*/*v*) dry cells, was mixed with 3% (*w*/*v*) sodium alginate solution at 1:2 (*v*/*v*). The mixture was extruded through syringe needles into a 0.1 M CaCl_2_ solution. The resulting beads were cured for 2 h and then hardened overnight at 4 °C. Optimum operation conditions were achieved with 770 g sucrose/L, fed at 200 L/h at 50 °C, and allowed for a productivity of 180 g FOS /L h. Moreover, the initial activity was maintained for about 100 days of operation in a 1.2 m^3^ reactor, so that a FOS content of 57% (*w*/*w*) in the effluent was obtained throughout this period. *Aureobasidium pullulans* cells were mixed with an alginate solution 3% (*w*/*v*), and the mixture was extruded into a 1% (*w*/*v*) CaCl_2_ solution to form small gel beads as hydrated-immobilized cells [142]. The immobilized cells were cured at room temperature for 2 h and then hardened overnight at 4 °C. The beads were then placed at −15 °C for 6–24 h to induce freeze-dehydration. The freeze-dehydration resulted in shrinkage of the beads as a result of water removal. As a result, the bead volume was reduced by 82% and the bead weight by 85%. The dehydrated beads were successfully used for the production of FOS in a model reactor system.

Other techniques have been studied for enzyme and cell immobilization to produce FOS. A crude β-fructofuranosidase preparation from *Aspergillus awamori* NBRC4033 was covalently bound to chitosan by glutaraldehyde linkages and used for FOS synthesis from sucrose [143]. The obtained crude extract, after filtration and centrifugation, was directly used as an enzyme source in the immobilization process. This involves firstly the solubilization and activation of chitosan, respectively, with acetic acid and glutaraldehyde at 1% (*v*/*v*), followed by the covalent binding of the enzyme to the GA-activated chitosan at pH 5.0. In the conditions used, 88% of the crude extract was bound, and an activity recovery of 54% was reported. No significant changes were observed in the pH and temperature optima of the immobilized biocatalyst, as compared to the free form, yet both pH and thermal stability of the former were increased. Immobilization resulted in diffusion limitations, as reflected by a 1.3-fold increase in *K*_m_. Transfructosylation only occurred at high sucrose concentrations, from 133.35 to 231.94 g/L, while at lower sucrose concentrations, 51.2 to 74.29 g/L, hydrolysis took place. The maximum conversion yield of sucrose to FOS was 55%, corresponding to 128 g/L of FOS and to a calculated productivity of 18.53 g/h/100 g. Kovaleva *et al.* [144] have shown that inulinase covalently immobilized on macroporous anion-exchange resin (AV-16-GS) had higher optimal temperature and pH stability than free inulinase. Commercial pectinase preparations with fructosyltransferase activity, Pectinex Ultra SP-L, have also been immobilized in Sepabeads EC-EP3 and EC-EP5 carriers and applied to the batch synthesis of fructooligosaccharides. A sufficiently high sucrose concentration, 630 g/L, was used to shift activity towards transfructosylation, in detriment of hydrolysis [145]. The reaction was faster using EC-EP5 carriers, possibly because of reduced diffusion limitations presented by this support, given its morphology. The FOS concentration reached a maximum value of 387 g/L after 36 h (240 g/L GF_2_, 144 g/L GF_2_ and 3 g/L GF_4_), which corresponds to 61.5% (*w*/*w*) of the total carbohydrates in the mixture. The features of these immobilized biocatalysts are very attractive for their application in batch and fixed-bed bioreactors. The fructosyltransferase produced by *Rhodotorula* sp. was immobilized in an inorganic support consisting of a niobium and graphite alloy [146]. The immobilization of the enzyme was by ionic binding to the surface of the particles, since the support is a charged and compact solid, with negligible porosity. The immobilized fructosyltransferase in niobium allowed for FOS yields of around 46% from sucrose concentrations of 50% and 70% (*w*/*v*), after 96 h of synthesis. GF_2_ was the predominant fructan produced, with GF_3_ and GF_4_ present in smaller amounts. A predictive model of FOS production was developed, which provided a close fit to experimental data.

## 6. Other Immobilized Carbohydrases

### 6.1. Dairy Products

The enzyme β-galactosidase (3.2.1.23) is classified as a hydrolase, with transferase capacity for galactosyl groups, catalyzing the hydrolysis of the terminal residue β-galactopyranosyl from lactose (Gal β 1–4Glc) to form an isomolecular mixture of glucose and galactose [20]. β-Galactosidase is an important enzyme in the food industry and has found significant applications in enhancing sweetness, solubility, flavor and digestibility of dairy products [147].

Guidini *et al.* [148] studied the immobilization process of *Aspergillus oryzae* β-galactosidase using the ionic exchange resin Duolite A568 as a carrier for lactose hydrolysis. The immobilization process by ionic binding was studied through a central composite design (CCD). The simultaneous influences of the enzyme concentration and pH on the immobilization medium were analyzed by the authors. The results showed that the retention of enzymatic activity during the immobilization process was strongly dependant on those variables, being maximized at pH 4.5 and an enzyme concentration of 16 g/L. The immobilized enzyme obtained under the previous conditions was subjected to a cross-linking process with glutaraldehyde, and the conditions that maximized the activity consisted of a glutaraldehyde concentration of 3.83 g/L and a cross-linking time of 1.87 h. The glutaraldehyde cross-linking increased the initial activity, retaining 90% of its initial activity. The authors related that the enzyme was very stable on pH ranges of 3.5–8.0, being appropriate for application in products with low pH values, such as acid whey, since the enzyme activity was maximized at pH 4.5.

Pessela *et al.* [149] performed the immobilization of β-galactosidase from *Thermus* sp. T2 via ionic adsorption onto two different supports: a new anionic exchanger resin, based on the coating of Sepabeads internal surfaces with polyethylenimine (PEI) polymers (Mw 25,000), and traditional DEAE-agarose. The adsorption strength was much higher in the case of PEI-Sepabeads than in DEAE-supports at both pH 5 and 7. Less than 5% of enzyme was eluted from PEI-support, while more than 70% protein was eluted from DEAE-agarose at pH 7 and 0.4 M NaCl. The PEI-derivatives remained almost fully active at pH 5 and 7 after several weeks of incubation at 50 °C, and their thermal stability was enhanced as compared to that of the free form. In particular, the derivative was used to perform the hydrolysis of 5% lactose solution for 50 h at pH 7 and 50 °C, with no noticeable shift in the pattern of the reaction. Moreover, it was established that after such a cycle, the derivative could be fully removed from the support and the latter re-loaded. Given these features, the authors suggested the immobilized biocatalyst presents a promising candidate for industrial use as a biocatalyst in the hydrolysis of lactose in different dairy products and could be coupled with the antimicrobial treatment usually performed. Moreover, Pessela and co-workers were able to establish that covalent immobilization of β-galactosidase from *Thermus* sp. T2 on boronate-epoxy-Sepabeads and on chelate-epoxy-Sepabeads reduced significantly the inhibition by glucose (non-competitive) and by galactose (competitive) observed for the free form [150]. At the same time, the *K*_m_ for lactose only increased by two-fold as an outcome of immobilization. The authors associated these outcomes to small modifications in the active center conformation that may have occurred after immobilization onto the heterofunctional epoxy-Sepabeads, which led to a more marked decrease in the affinity of the enzyme to the inhibitor than that to the substrate. Thus, using either immobilized enzyme preparation for the hydrolysis of a 5% (*w*/*v*) lactose solution, the reaction proceeded up to a hydrolysis yield over 99%, whereas for the free enzyme, only 85% hydrolysis could be achieved.

A rather similar observation was reported by Mateo and co-workers when immobilizing β-galactosidase from *Kluyveromyces lactis* in agarose-glyoxyl, glutaraldehyde-agarose and glutaraldehyde-Eupergit C supports [151]. Yet, in these supports, only the non-competitive inhibition by glucose was significantly reduced, while both the affinity of the enzyme to the competitive inhibitor (galactose) and to the substrate (lactose) remained unaltered, as compared to the free form, possibly due to steric hindrances to the access of glucose to the binding site. Somehow, curiously, the immobilization to an Eupergit-boronate derivative led to no changes in the non-competitive inhibitor. This was considered the result of binding taking place through the sugar chains, thus leaving the inhibition site exposed. Actually, no modifications were noticed regarding the affinity of the enzyme to lactose and galactose as a result of immobilization. Moreover, the glyoxyl-agarose derivative was for 20 reactions with a marginal decrease enzyme activity and allowing for almost full hydrolysis of a 5% (*w*/*v*) lactose solution at the end of the final batch.

Tanriseven and Dogan [152] immobilized β-galactosidase from *Aspergillus oryzae* in fibers composed of alginate and gelatin cross-linked with glutaraldehyde to avoid leaking out of the enzyme. The immobilization yield was of 56%, and the activity of the immobilized biocatalyst remained stable for 35 days. The optimum pH and temperature were not affected by immobilization and remained at 4.5 and 50 °C, respectively. Yet, higher activity retention at extreme pH and temperature was observed for immobilized β-galactosidase. A 1.2-fold increase in *K*_m_ as a result of immobilization suggests a typical diffusion limitations increase, and the maximum specific reaction rate decreased almost by half, suggesting structural changes in the enzyme. β-galactosidase was immobilized by adsorption on a mixed-matrix membrane containing zirconium dioxide [153]. Up to 1.6 g β-galactosidase could be absorbed per m^2^ of membrane. However, maximal activity was achieved at an enzyme load of around 0.5 g/m^2^. Although the optimum pH for activity was shifted from 6.5 to 7.0, due to immobilization, no significant change was observed in the optimum temperature. Activity retention was, however, more effective at lower temperatures, within the range tested (5 to 40 °C). Moreover, an eight-fold increase in the apparent *K*_m_ was observed after immobilization, although *V*_max_ remained unaltered. The authors suggest the feasibility of the immobilized biocatalysts for application to GOS synthesis.

### 6.2. Isomaltulose Production

Isomaltulose is a monosaccharide that presents remarkable applications in the food industry. This sugar is an interesting substitute for sucrose, considering that it is approximately 50% as sweet as sucrose [154], it is non-carcinogenic [155] and it has a low glycemic index [156]. In addition, isomaltulose can be converted into sugar alcohol, Isomalt^®^, which has other useful properties for foods. Isomaltulose can be industrially obtained by transglycosylation, catalyzed by the intracellular glucosyltransferase produced by some bacteria strains.

There are several works on the immobilization of bacterial cells to produce isomaltulose. Yet, considering the advantages of the use of enzyme extracts, mainly the reduced level of microbial contamination of the product, the immobilization of the extracted glucosyltransferase can be attractive if an efficient immobilization method is developed.

In the work of Kawaguti and Sato [8], isomaltulose production was studied in a repeated-batch process using immobilized glucosyltransferase. The highest conversion rate of sucrose into isomaltulose was obtained in the first batches, being 43.1%. It was observed that after the first batch, the conversion rates decreased quickly, and in the second batch, an isomaltulose production rate of 7.3% was obtained. The same research group reported the immobilization of glucosyltransferase extracted from the cells of *Erwinia* sp. onto Celite 545. The authors observed that the optimal conditions for immobilization were at pH 4.0 and 170 U of glucosyltransferase/g of Celite 545. Using these conditions, more than 60% conversion of sucrose into isomaltulose could be obtained. The enzyme was also immobilized in microcapsules of low-methoxyl pectin and fat (butter and oleic acid). The non-lyophilized microcapsules of pectin containing the glucosyltransferase and fat converted 30% of sucrose into isomaltulose in the first batch [157].

## 7. Conclusions

As reported in this review, different types of carbohydrases have huge potential for application in the food industry. However, because of the cost for the application of these biocatalysts, many studies should focus on the development of cheap and efficient immobilization methods. Considering that the application is for the food industry, different parameters are considered. For example, most of the steps where these enzymes are applied are in an aqueous solution. Thus, if the enzyme is weakly adsorbed into the support, it can easily desorb from the matrix. On the other hand, if adsorption/desorption occurs in differentiated and well-controlled environments, carrier reuse, once enzymatic activity is exhausted, is feasible. Unwanted enzyme desorption can be avoided with the covalent bonding of the enzyme into the support. But, extreme caution should be taken considering that this technique needs extremely toxic reagents, such as glutaraldehyde. Significant advances have been made in this field with the introduction of pre-activated hetero-functional supports, which somehow simplify the methodology and, moreover, allow the site-directed rigidification of the enzyme. The feasibility of the method requires, nevertheless, knowledge of the residues of the catalytic site to prevent drawbacks similar to those in random binding. Moreover, extreme rigidification may be a drawback for enzymes displaying large conformational changes during catalysis. Finally, the entrapment of the enzyme into some gels or synthetic polymers has to be very well studied, taking into consideration the porosity of the matrix and the size of the enzyme (molecular weight) and of the substrate/product. However, if adequately identified and implemented, the immobilization methods can enhance enzyme stability, activity and selectivity. In the design of the immobilization methods, it should be brought to mind that the selection of the immobilization method must take in to consideration the specific nature of the reaction to be catalyzed and the reactor configuration and not only consider the highest stabilization and activity of the enzyme. This is of relevance, since successful industrial applications of the immobilized biocatalysts require not only that it is stable and active, but also that it has low cost, can undergo repeated re-use and is compatible with the intended set-up. Growing knowledge on the 3D structure of enzymes and in their surface composition provides a powerful tool for a more rational design of immobilization, but this has to be combined with insight on the physical and chemical properties of the support. The application of molecular simulation methods has been contributing to the rational development of immobilization strategies, but this approach is still limited. Further experimental evidence on the structure of the enzyme after immobilization is still required to better understand the outcome of immobilization and, thus, help the development of suitable models. Given the large number of enzymes with interest for the food and feed industry, along with that of promising available supports, and the vast resources required for their molecular characterization and development of predictive models, it can be foreseen that the random, trial and error approach will still be used in the near future, at least in a screening role. The current high throughput platforms currently available allow for the evaluation of a large number of variables in a timely, cost-effective manner. A major challenge is also the development of tailor-made carriers displaying tunable binding, geometric and mechanical properties, so that they can be used in different reactor configurations and, so, allow a more efficient and cost effective continuous use or reuse of the biocatalysts. The successful accomplishment of such an endeavor is clearly a multidisciplinary task, since it involves computational modeling of materials, life science and computational scientists, alongside with biochemical engineers

## Figures and Tables

**Table 1:** Biotechnological applications of microbial carbohydrases in the food industry.

Carbohydrase	Microbial source	Application	Reference
*Amylases*	*Bacillus megaterium VUMB109*	Maltooligosaccharides production	[2]
Invertases	*Sacharomyces cereviseae*	Hydrolysis of sucrose	[3]
Inulinases	*Aspergillus niger*	Fructose syrup production	[4]
β-galactosidase	*Lactobacillus delbrueckii subsp. bulgaricus ATCC 11842*	Lactose hydrolysis in milk	[5]
	*Aspergillus oryzae*	Synthesis of galactooligosaccharides	[6]
β-glucosidase	*Rhizomucor miehei NRRL 528*	Enhancement of the amounts of free phenolic antioxidants in sour cherry pomace	[7]
Glucosyltransferase	*Erwinia sp.*	Isomaltulose production	[8]

**Table 2:** Carbohydrases immobilization using different immobilization techniques.

Enzyme	Support	Immobilization method	Improvement compared to the free form	Reference
α-amylase	Functionalized glass beads	Covalent binding	Better thermostability and reuse after six runs	[15]
Glucoamylase	Polyaniline polymer	Covalent binding	Better thermostability and higher stability in the alkaline range	[16]
Pullulanase	Magnetic chitosan beads	Covalent binding	Higher relative activity, stabilization of enzyme over a broader pH range	[17]
Inulinase	DEAE-Cellulose	Adsorption	Better thermostability	[18]
Invertase	Calcium alginate gel capsules	Entrapment	Better stability at high pH and temperatures	[19]
β-Galactosidase	Polysiloxane–polyvinyl alcohol magnetic (mPOS–PVA) composite	Covalent binding	Higher operational and thermal stability	[20]
β-glucosidase	Eupergit C	Covalent binding	Improvement of the stability	[21]
Pectinase	Sodium alginate support using glutaraldehyde	Covalent binding	Higher thermostability and reusability	[22]
Fructosyltransferase	Eupergit C	Covalent binding	Reuse for 20 batch reactions, better stability at high pH and temperatures	[23]
